# Supplementary material for: Content-rich biological network constructed by mining PubMed abstracts
Source: BMC Bioinformatics. 2004 Oct 8;5:147. doi: 10.1186/1471-2105-5-147 (PMC528731; doi:10.1186/1471-2105-5-147)
Supplement: Additional File 5 — The original Chilibot query results of the term "long-term potentiation (LTP)" and 22 other terms, limiting the latest references analyzed to the years 1990, 1995, 2000, and 2004. [file 1471-2105-5-147-S5.bz2 › chilibotAdditionalFile5/ltp2000/html/PKA_AMPA.html]

 


 **PKA** and **AMPA** 
  
Found 37 abstracts in PubMed,  **30 abstracts were retrieved and analyzed**.  


---

 Search Google  |
 PDF files only 
|  EDU domain only 

---

**Interactive relationship** (e.g. stimulation, inhibition, etc)

- **PKA**  activators inhibited both  **AMPA**  receptor dephosphorylation and LTD.  Ref: 9856471 Neuron, 1998
- Also, it seems that 1 electronegative group substitutions on the isoxazole ring of  **AMPA**  and 2 decreasing the  **pKa**  of the sub stituent at position 3 play a major role in determining the degree of receptor activation under steady state conditions.  Ref: 9272640 J Neurosci Res, 1997
- Training in step down inhibitory avoidance 0.3 mA footshock is followed by biochemical changes in rat hippocampus that strongly suggest an involvement of quantitative changes in glutamate  **AMPA**  receptors, followed by changes in the dopamine D1 receptor cAMP protein kinase A  **PKA**  CREB P signalling pathway in memory consolidation.  Ref: 9361725 Braz J Med Biol Res, 1997
- In addition, the modulation of GluR1 by  **PKA**  phosphorylation of Ser 845 suggests that phosphorylation of this residue may underlie the  **PKA**  induced potentiation of  **AMPA**  receptors in neurons.  Ref: 8663994 Neuron, 1996
- We find that synthesis of  **AMPA**  receptor subunits is increased three hours after LTP induction this effect on the synthesis of the  **AMPA**  receptor is blocked by inhibitors of  **PKA**  and of transcription.  Ref: 9716131 Nature, 1998
- These results suggest that  **AMPA**  receptor peak response open probability can be increased by  **PKA**  through phosphorylation of GluR1 Ser845.  Ref: 10627585 J Neurosci, 2000
- Cell based studies indicate that phosphorylation of  **AMPA**  receptors is enhanced by a SAP97 AKAP79 complex that directs  **PKA**  to GluR1 via a PDZ domain interaction.  Ref: 10939335 Neuron, 2000
- Our results support the idea of a postsynaptic mechanism maintaining late phase LTP, in which  **AMPA**  receptor synthesis is increased as a result of  **PKA**  dependent gene transcription.  Ref: 9716131 Nature, 1998
- Others have shown that alpha amino 3 hydroxy 5 methyl 4 isoxazole propionate  **AMPA**  type glutamate receptor mediated current is potentiated by cyclic AMP dependent protein kinase  **PKA**  activation.  Ref: 10582604 J Neurochem, 1999
- Recent studies have shown that phosphorylation of  **AMPA**  receptors by cAMP dependent protein kinase  **PKA**  and calcium and calmodulin dependent protein kinase II CaMKII potentiates their activity, but phosphorylation of the receptor subunits may also affect their interaction with intracellular proteins, and their expression at the plasma membrane.  Ref: 11059799 Neurochem Res, 2000
- Hippocampal N methyl D aspartate NMDA receptor dependent long term synaptic depression LTD is associated with a persistent dephosphorylation of the GluR1 subunit of  **AMPA**  receptors at a site Ser 845 phosphorylated by cAMP dependent protein kinase  **PKA** .  Ref: 9856471 Neuron, 1998
- These results suggest that dephosphorylation of  **AMPA**  receptors is an expression mechanism for LTD and indicate an unexpected role of  **PKA**  in the postsynaptic modulation of excitatory synaptic transmission.  Ref: 9856470 Neuron, 1998

**Parallel relationship** (e.g. studied together, co-existance, homology, etc.)

- Our data demonstrate that activation of D1 type dopamine receptors on striatal neurons promotes phosphorylation of  **AMPA**  receptors by  **PKA**  as well as potentiation of current amplitude.  Ref: 10582604 J Neurochem, 1999
- No simple correlation between activity as  **AMPA**  agonists in vitro and  **pKa**  values of these compounds was apparent.  Ref: 9046343 J Med Chem, 1997
- **AMPA**  and kainate activated receptors are modulated by adenosine monophosphate cAMP dependent protein kinase A  **PKA**  coupled via D1 dopamine receptors.  Ref: 10331215 Naturwissenschaften, 1999
- PURPOSE This study was undertaken to test the hypothesis that topiramate TPM exerts a negative modulatory effect on some types of alpha amino 3 hydroxy 5 methylisoxazole 4 proprionic acid  **AMPA**  kainate receptors by binding to the site at which protein kinase A  **PKA**  phosphorylates the receptor channel complex.  Ref: 10768301 Epilepsia, 2000
- Inclusion of purified catalytic subunit Calpha  **PKA**  in the pipette solution increased neuronal  **AMPA**  receptor P compared with recordings made with calcineurin included in the pipette 0.39.  Ref: 10627585 J Neurosci, 2000
- We have examined the effect of  **PKA**  and calcineurin on the time course, peak open probability, and single channel properties of glutamateevoked responses for neuronal  **AMPA**  receptors and homomeric GluR1 receptors recorded in outside out patches.  Ref: 10627585 J Neurosci, 2000
- Ten minutes prior to the retention test, through indwelling cannulae placed in the CA1 region of the dorsal hippocampus, they received 0.5 microl infusions of saline, a vehicle 2% dimethylsulfoxide in saline, the glutamate NMDA receptor blocker, aminophosphonopentanoic acid AP5 5.0 microg, the  **AMPA**  kainate receptor blocker, cyanonitroquinoxaline dione CNQX 0.25 or 1.25 microg, the metabotropic receptor antagonist, methylcarboxyphenylglycine MCPG 0.5 or 2.5 microg, the inhibitor of calcium calmodulin dependent protein kinase II KN62 3.5 microg, the inhibitor of cAMP dependent protein kinase  **PKA** , Rp cAMPs 0.1 or 0.5 microg, the stimulant of the same enzyme, Sp cAMPs 0.1 or 0.5 microg, or the inhibitor of the mitogen activated protein kinase MAPK kinase, PD098059 10 or 50 microM.  Ref: 10840135 Behav Brain Res, 2000
- Using phosphorylation site specific antibodies, we found that induction of chem LTD produces a persistent dephosphorylation of the GluR1 subunit of  **AMPA**  receptors at serine 845, a cAMP dependent protein kinase  **PKA**  substrate... a substrate of protein kinase C PKC and calcium calmodulin dependent protein kinase II CaMKII.  Ref: 9856470 Neuron, 1998
- H  **AMPA**  IC50 = 2 microM, and 5 nitro DL willardiine  **pKa**  6.  Ref: 9379437 J Med Chem, 1997
- Maintenance of late phase LTP is accompanied by  **PKA**  dependent increase in  **AMPA**  receptor synthesis.  Ref: 9716131 Nature, 1998
- 0 or 5.0 microg, the  **AMPA**  receptor blocker, dinitroquinoxaline 1H, 4H dione DNQX, 0.4 or 1.0 microg, the metabotropic receptor antagonist, methylcarboxyphenylglycine MCPG, 0.5 or 2.5 microg, the inhibitor of cAMP dependent protein kinase  **PKA** , Rp cAMPs 0.1 or 0.5 microg, the  **PKA**  stimulant, Sp cAMPs 0.5 microg, or the inhibitor of the mitogen activated protein kinase MAPK, PD098059 10 or 50 microM.  Ref: 10996059 Behav Brain Res, 2000
- 0 microg, the  **AMPA**  receptor blocker, cyanonitroquinoxaline dione CNQX, 1.25 microg, the generic glutamate metabotropic receptor antagonist, alpha methyl 4 carboxyphenyl glycine MCPG, the inhibitor of cAMP dependent protein kinase  **PKA** , Rp cAMPs 0.1 or 0.5 microg, or the inhibitor of the mitogen activated protein kinase MAPK, PD098059 10 or 50 microM.  Ref: 11099775 Behav Brain Res, 2000
- These findings suggest that dephosphorylation of a  **PKA**  site on  **AMPA**  receptors may be one mechanism for NMDA receptor dependent homosynaptic LTD expression.  Ref: 9856471 Neuron, 1998
- The results indicate that the effect of novelty on retrieval can not be observed if the major biochemical mechanisms of retrieval  **AMPA**  receptors,  **PKA** , MAPK are blocked, i.e. if the hippocampus was temporarily inactivated by drugs that inhibit those mechanisms.  Ref: 11099775 Behav Brain Res, 2000
- The results correlate with pharmacological findings showing an early post training involvement of  **AMPA**  receptors, and a late involvement of the D1 cAMP  **PKA**  CREB P pathway in memory consolidation of this task.  Ref: 9361725 Braz J Med Biol Res, 1997
- The phosphorylation of the glutamate receptor 1 GluR1 subunit of  **AMPA**  receptors by protein kinase A  **PKA** , protein kinase C PKC, and calcium calmodulin dependent protein kinase II CaMKII has been characterized extensively.  Ref: 10366608 J Neurosci, 1999
